# Clinical utility of FDG uptake within reticuloendothelial system on F-18 FDG PET/CT for prediction of tumor recurrence in breast cancer

**DOI:** 10.1371/journal.pone.0208861

**Published:** 2018-12-07

**Authors:** Ji-In Bang, Hai-Jeon Yoon, Bom Sahn Kim

**Affiliations:** Department of Nuclear Medicine, Ewha Womans University School of Medicine, Seoul, South Korea; Ente Ospedaliero Cantonale, SWITZERLAND

## Abstract

**Background:**

The aim of this study was to investigate the metabolism of the spleen, bone marrow (BM), and liver from preoperative F-18 FDG PET/CT scans for the prediction of recurrence in breast cancer.

**Methods:**

We retrospectively included 153 patients diagnosed with invasive ductal carcinoma (IDC) of the breast who underwent preoperative F-18 FDG PET/CT scan and a curative operation. The mean standardized uptake value (SUV_mean_) of the spleen, liver, and BM and maximum SUV (SUV_max_) of primary tumors were measured. The relationships between spleen, BM, and liver metabolism and clinicopathologic parameters were evaluated, and possible prognostic parameters predicting recurrence were assessed using disease-free survival (DFS).

**Results:**

Spleen SUV_mean_ was significantly correlated with primary tumor SUV_max_, pathologic T (pT) stage, and histologic grade of primary tumor. BM SUV_mean_ also showed a positive correlation with primary tumor SUV_max_. Spleen SUV_mean_ were significantly associated with recurrence from binary logistic regression analysis (*P* = 0.004). Spleen, BM, liver, and primary tumor SUVs were all significant prognostic factors for DFS in univariate Cox regression analysis (all *P*<0.024). Among all PET parameters analyzed, spleen SUV_mean_ ≥ 2.21 (*P* = 0.032) was in the multivariable analysis the powerful poor prognostic factor predicting DFS that was independent of other clinicopathological features like T stage (pT >2; *P* = 0.009) and estrogen receptor (ER) status (ER negativity; *P* = 0.001).

**Conclusion:**

Splenic metabolism together with pT stage and ER status was an independent prognostic factor for predicting recurrence in breast cancer. Metabolic activity of reticuloendothelial system such as spleen, liver or BM on preoperative F-18 FDG PET/CT can be a meritorious imaging factor for discriminating patients with IDC that require adjunctive therapy to prevent recurrence.

## Introduction

In recent years, the systemic inflammatory response to cancer has received growing interest as an important factor in cancer development and prognosis [1–4]. Consistent evidence indicates that a systemic inflammatory response in cancer is related to the prognosis of various cancers [5]. Therefore, it is anticipated that assessing the systemic inflammatory response in cancer patients could be useful as a stratification marker for oncologic clinical practice. Biomarkers such as neutrophil-to-lymphocyte ratio (NLR), platelet-to-lymphocyte ratio (PLR), C-reactive protein (CRP), and cytokines have been suggested as measurable parameters of systemic inflammation and prognostic predictors in various cancers [3, 5, 6].

F-18 fluorodeoxyglucose (FDG) positron-emission tomography/computed tomography (PET/CT) has been widely used in oncology to evaluate the metabolism of tumors. There have been attempts to measure the systemic inflammatory response from F-18 FDG PET/CT. As F-18 FDG uptake indicates glycolytic activity, not only in tumorous conditions, but also under inflammation, the systemic inflammatory response can be detected and assessed through F-18 FDG PET/CT images. Because the metabolism of bone marrow (BM) is known to correlate with hematologic parameters [7, 8], there have been several reports that F-18 FDG uptake of BM could be useful for assessing the systemic inflammatory response as a predictor of prognosis in various cancers including lung cancer [9], stomach cancer [10], and colorectal cancer [11]. As components of the reticuloendothelial system (RES), spleen and liver also have been considered integral parts of the systemic inflammatory response in cancer [12]. Activation of RES organs can be measured by metabolic activity using F-18 FDG PET/CT [13]. Metabolic activity of the spleen is known to be an independent prognostic factor in lung cancer and cholangiocarcinoma [14, 15]. Also, previous study from stomach cancer patients showed that splenic metabolism might have a predictive role in prognosis [16]. Therefore, the metabolic activity of BM and spleen on F-18 FDG PET/CT could be a promising prognostic indicator in various type of cancer patients.

In breast cancer, the pretreatment values of NLR and PLR are known predictors of mortality [17–20]. Recently, metabolism of RES organs on F-18 FDG PET/CT showed significant correlation with clinicopathologoical features in breast cancer patients [21]. However, the clinical prognostic significance of the F-18 FDG uptake of spleen, BM, or liver has not been assessed in patients with breast cancer. In the present study, we investigated the relationships between F-18 FDG uptake of the spleen, BM, and liver on F-18 FDG PET/CT with clinicopathological parameters and assessed the possible prognostic value of the F-18 FDG parameters in predicting disease-free survival (DFS) in patients with breast cancer.

## Materials and methods

All procedures were in accordance with the principles of the 1975 Declaration of Helsinki (2013 revision). Study design and exemption from informed consent were approved by the Institutional Review Board of Mokdong Hospital of Ewha Womans University (No.2018-04-036).

### Patients

We retrospectively reviewed patients who were histopathologically diagnosed with invasive ductal carcinoma (IDC) of the breast and underwent a preoperative F-18 FDG PET/CT scan for initial staging procedure between November 2010 and April 2014 in our medical center. Patients who underwent neoadjuvant chemotherapy before the operation due to clinically stage of T4 (cT4) or over N2 (cN2)were not included. In addition, in case of below cT3 or cN1, patients who underwent neoadjuvant chemotherapy for purpose of breast-preservation were not included. Among the eligible patients, we excluded those who (i) had tumors <1 cm in size to minimize a partial-volume averaging effect in analysis of F-18 FDG PET/CT images, (ii) had a previous history of any other malignancy or other malignancy that developed during follow-up, (iii) were lost to follow-up before 6 months after surgery without any event, (iv) refused to receive adjuvant therapy after surgery, (v) had any liver disease including chronic viral infection (hepatitis B or C) or abnormal liver function test (LFT) results in a preoperative assessment, and (vi) had history of recent administration of medication such as granulocyte-colony stimulating factor (G-CSF). We also reviewed the electronic medical record to reveal any clinical symptom or sign other than breast cancer at the time of preoperative work-up for possible other underlying disease.

### Clinicopathological and survival data

Clinicopathological data that were considered potentially relevant for prognosis were collected from electronic medical records. Data included age at initial diagnosis, sex, menopausal status, body mass index (BMI) defined as the ratio of body weight in kilograms to the square of height in meters, and blood tests results as preoperative work-up including whole blood cell count, platelets, and hemoglobin (Hb). NLR and PLR were calculated using preoperative blood cell count results. Pathologic data included pathologic stages (pT, pN) based on the 7^th^ American Joint Committee on Cancer staging system [22], pathologic tumor diameter, surgical margin involvement, Black’s nuclear grade, and Bloom-Richardson’s histologic grade. Immunohistochemical results evaluating the expression of estrogen receptor (ER), progesterone receptor (PR), human epidermal growth factor receptor 2 (HER2), and Ki67 were also collected. ER and PR positivity statuses were defined using both scoring parameters of the percentage of positive cells (from 0 to 5) and staining intensity (from 0 to 3). A total score of 0–2 was considered negative for each hormone, and 3–8 was considered positive. HER2 was defined as negative when the immunohistochemistry results were negative or 1+ and positive when the results were 3+. When the results were 2+, the positivity of HER2 was defined according to the results of fluorescence in situ hybridization (FISH). For the marker Ki67, overexpression was defined as present in more than 14% of the tumor cells.

LFT results including liver transaminase (aspartate aminotransferase, AST, and alanine aminotransferase, ALT), total bilirubin, and serologic evaluation for hepatitis B and C as preoperative work-up were included.

After curative surgery, adjuvant therapy was performed according to pathologic staging and clinical condition of the patients. Regular clinical follow-up was conducted with physical examination, blood test, ultrasonography, chest X-ray, and bone scan every 3 to 6 months in the first 2 years and every 6 to 12 months after that. When recurrence was suspected, additional procedures for pathological confirmation or imaging work-up were performed. For survival analysis, DFS was defined as the time between the date of operation and the date that any recurrence was first identified.

### Acquisition of F-18 FDG PET/CT

Acquisition of F-18 FDG PET/CT was done as described in detail previously [23]. All patients fasted for at least 6 h before the F-18 FDG PET/CT scans. Blood glucose level was measured and was required to be less than 140 mg/dL before intravenous injection of 5.18 MBq/kg of F-18 FDG. A CT scan was performed at 120 kVp without contrast enhancement for attenuation correction and anatomical information. A PET scan was obtained from the skull base to the upper thigh, using a Siemens Biograph mCT with 128-slice CT (Siemens Medical Solutions, Erlangen, Germany) at 1 h (mean 60.7 min, range 51–79 min) after FDG injection. The spatial resolution at the center of the PET was 2.0 mm full width at half maximum (FWHM) in the transaxial direction and 2.0 mm FWHM in the axial direction. Three-dimensional emission was used for the acquisition parameters for PET images, and it required a 2 min scan per bed position for 5–7 positions. PET images were reconstructed to 200 × 200 matrices and 3.4 mm × 3.4 mm pixel sizes with a 3.0-mm slice thickness using a three-dimensional OSEM iterative algorithm (2 iterations and 21 subsets) with time of flight and point spread functions. The patients were in a supine position and allowed to breathe normally during PET/CT image acquisition.

### Analysis of F-18 FDG PET/CT imaging

All PET/CT data were analyzed using a commercially available system (Syngo.via; Siemens Medical Solutions, Erlangen, Germany). The maximal standardized uptake value (SUV_max_) corrected for the individual patient body weight for primary breast cancer lesion (primary tumor SUV_max_), and the mean SUVs (SUV_mean_) of spleen, liver, and BM were measured.

Regions of interest (ROI) were placed over the primary tumor using the automated delineation features in commercial software described in above. For spleen SUV_mean_, a spherical volume of interest (VOI) was drawn manually on the center of the spleen, which showed the largest anterior-posterior diameter on the axial image. To validate method of spleen SUV measurement, the inter-rater variability was assessed. Two nuclear medicine physicians who were strictly blinded to the patient’s clinical information measured the spleen SUVs. The metabolism of BM was measured using a method reported previously [9, 11, 24, 25]. A spherical VOI was drawn over the vertebral body of T10-T12 spines and L3-5 spines, and the SUV_mean_ was measured using the 75% cutoff value of the SUV_max_. Compromised spine, such as compression fracture, degeneration, or postoperative change, was omitted from BM metabolism measurement. The mean value of SUV_mean_ of BM was defined as BM SUV_mean_. For measuring the metabolism of the liver (liver SUV_mean_), a spherical VOI was automatically placed on the right of the liver using a fixed size (3 cm diameter). All automatically drawn reference VOIs were reviewed and manually redrawn if the CT scan quality was compromised or the liver was displaced by other lesions.

### Statistical analysis

First, intraclass correlation coefficient was used to assess the inter-rater variability in spleen SUV measurements. To evaluate the relationships between metabolism of spleen, BM, liver and clinicopathological parameters, Spearman’s rank correlation analyses were employed. Clinicopathological and PET parameters were analyzed using binary logistic regression for possible prediction of recurrence. Survival analysis was performed using clinicopathological and PET parameters as covariates and tumor recurrence as endpoints. DFS was calculated using the Kaplan-Meier method. Univariate Cox regression analysis was performed for each parameter and variable, and those with a P value less than 0.05 were included in the multivariable analysis. For multivariable Cox regression, the forward conditional method (entry threshold P<0.05; removal threshold P>0.1) was applied. For continuous variables, optimal cutoff points for parameters were determined by finding the point with the most significant (log-rank test) split of DFS, using web-based R software engineered and designed by Budczies et al. [26] (http://molpath.charite.de/cutoff/). Receiver operating characteristic (ROC) curves were generated, and area of under the ROC (AUC) for each optimal cutoff point were calculated to assess the predictive performance of recurrence. All statistical analyses were performed using a commercial statistical software package (SPSS Ver. 19.0; SPSS Inc., Chicago, IL), and two-sided P-values < 0.05 were considered statistically significant. For ROC analyses, GraphPad software (San Diego, CA) was used.

## Results

### Patient characteristics

We initially enrolled 226 female patients who fulfilled the inclusion criteria. Eleven patients (over cN2, n = 8; for breast-preservation, n = 3) were excluded due to neoadjuvant therapy. A total of 62 patients were excluded from final analysis due to tumor size <1 cm (n = 29), short follow-up period (n = 10), underlying malignancy other than breast cancer (n = 12), refusal of adjuvant therapy (n = 5), and underlying liver disease or abnormal LFT results (n = 6). The final analysis included 153 patients. Table 1 summarizes the demographics and tumor characteristics of the 153 enrolled patients. None of those enrolled patients received the G-CSF at the time of preoperative work-up or revealed any acute clinical symptom or sign other than breast cancer.

**Table 1:** Characteristics of the 153 enrolled patients.

Characteristics	Number	%
Sex		
Female, n (%)	153	100
Age (years) at initial diagnosis, mean±SD (range)	49.5±9.6 (30–79)	
Menopausal status		
Premenopausal	105	68.6
Postmenopausal	48	31.4
Operation		
Breast-conserving surgery	130	85.0
Mastectomy	23	15.0
pT stage		
T1	90	58.8
T2	61	39.9
T3	2	1.3
pN stage		
N0	107	69.9
N1	36	23.5
N2	9	5.9
N3	1	0.7
ER status		
Positive	104	68.0
Negative	49	32.0
PR status		
Positive	105	68.6
Negative	48	31.4
HER2 status		
Positive	40	26.1
Negative	113	73.9
Ki67 index status^‡^		
<14%	53	36.3
≥14%	93	63.7
Resection margin involvement		
Negative	150	98.0
Positive	3	2.0
Nuclear grade		
Grade 1	6	3.9
Grade 2	87	56.9
Grade 3	60	39.2
Histologic grade		
Grade 1	28	18.3
Grade 2	68	44.4
Grade 3	57	37.3
Pathologic Stage^†^		
I	74	48.4
II	68	44.4
III	11	7.2
Adjuvant Treatment		
CTx+RTx+HTx	111	72.5
CTx+RTx	10	6.5
RTx+HTx	13	8.5
CTx+HTx	16	10.5
CTx only	1	0.7
RTx only	1	0.7
HTx only	1	0.7
WBC^§^, mean±SD (range) (x 10^12^ cells/L)	6.05±1.50 (3.03–10.00)	

*ER* estrogen receptor, *PR* progesterone receptor, *HER2* human epidermal growth factor receptor 2, *CTx* chemotherapy, *RTx* radiation therapy, *HTx* hormonal therapy, *WBC* white blood cell

^‡^Ki67 value for 146 patients

^†^Pathologic staging according to the 7^th^ AJCC

^§^ Reference ranges for WBC count in normal adults is 4.00–10.00 x 10^12^ cells/L

Among the 153 patients, 19 (12.4%) had tumor recurrence. The mean clinical follow-up duration of the patients was 79.2 months (95% CI 75.4–83.1 months, range 8–88 months). Sites of tumor recurrence were locoregional sites (nine patients), lymph nodes (three patients), bone (two patients), brain (one patient), lung (two patients), liver (one patient), and multiple organs (one patient).

### Inter-rater variability in spleen SUV measurement

The intraclass correlation coefficient was 0.954, indicating the high reproducibility of t measuring the spleen SUV manually via spherical VOI on the center of spleen, which showed the largest anterior-posterior diameter on the axial image.

### Relationships between metabolism of BM, spleen, and liver and clinicopathologic parameters

**Fig 1:**
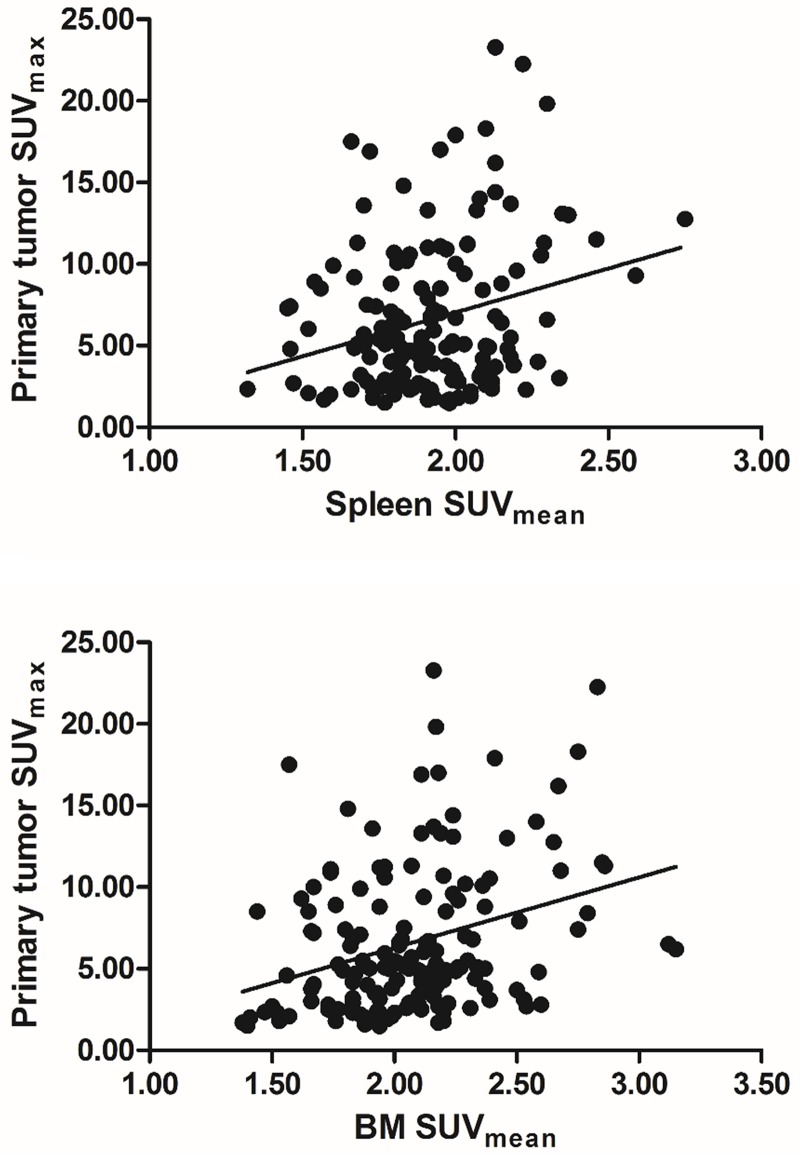
Scatter plot showing the relationships between primary tumor standardized uptake value (SUV)_max_ and (A) spleen SUV_mean_ and (B) bone marrow (BM) SUV_mean_.

Correlation analysis between the metabolism of spleen, BM, and liver and tumor-related parameters revealed that spleen SUV_mean_ was correlated significantly and positively with primary tumor SUV_max_ (rho = 0.187, *P* = 0.021, Fig 1A), pT stage (pT1 vs T2+3; rho = 0.165, *P* = 0.042), and histologic grade (grade 1 vs grade 2+3; rho = 0.212, *P* = 0.008). BM SUV_mean_ also showed a positive correlation with primary tumor SUV_max_ (rho = 0.323, *P*<0.001, Fig 1B).

Relationships between the metabolism of the spleen, BM, and liver and clinical parameters showed that the spleen, BM, and liver SUV_mean_ each had a positive correlation with BMI (rho = 0.551, *P*<0.001 for spleen SUV_mean_; rho = 0.224, *P* = 0.005 for BM SUV_mean_; rho = 0.434, *P*<0.001 for liver SUV_mean_). There was a significant negative correlation between BM SUV_mean_ and Hb (rho = -0.202, *P* = 0.012). However, spleen, BM, or liver SUV_mean_ was not correlated with any clinical parameters including WBC, NLR, and PLR.

### ROC curves analysis

**Fig 2:**
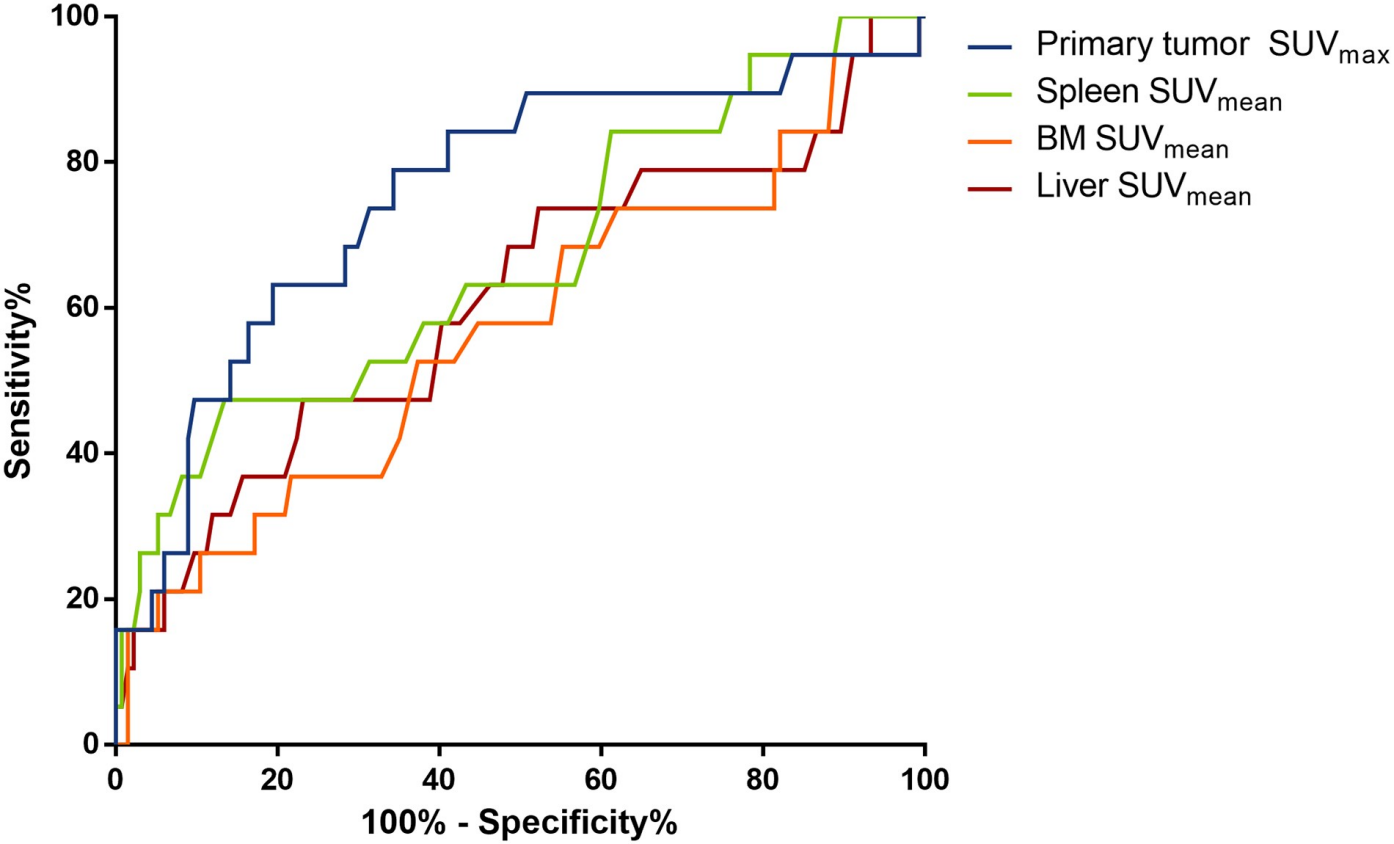
Receiver operating characteristic curves for primary SUV_max_, spleen SUV_mean_, BM SUV_mean_, and liver SUV_mean_.

The AUCs were 0.76, 0.67, 0.57 and 0.61 for primary tumor SUVmax, spleen, BM and liver SUVmean, respectively (Fig 2). Each optimal cutoff point determined by finding the point with the most significant (log-rank test) split of DFS were 11.27 (sensitivity 47.4%, specificity 90.3%), 2.21 (sensitivity 31.6%, specificity 94.8%), 2.625 (sensitivity 21.1%, specificity 94.8%), and 2.935 (sensitivity 21.1%, specificity 94%) for primary tumor SUVmax, spleen, BM and liver SUVmean, respectively.

### Recurrence and clinicopathologic, PET parameters

Binary logistic regression was performed to evaluate the possible clinicopathological and PET parameters for the prediction of recurrence. For clinical parameters, only BMI was significantly associated with recurrence. Pathological parameters including pT stage, lymph node metastasis, ER/PR status, tumor involvement of resection margin, nuclear grade, and histologic grade were significantly associated with recurrence.tumorAmong PET parameters, primary tumor SUV_max_ and spleen SUV_mean_ were significantly associated with recurrence. Table 2 summarizes the results of binary logistic regression associated with recurrence.

**Table 2:** Results of binary logistic regression to identify parameters associated with recurrence.

	Non-recur (n or mean±SD)	Recur (n or mean±SD)	P value	Odds ratio	95% CI
Age (years) at diagnosis	49.5±9.9	49.0±7.3	0.806	0.994	0.944–1.046
BMI	23.2±3.2	25.2±4.2	0.014	1.181	1.034–1.349
Menopausal status					
Premenopausal	94	11	-	-	-
Postmenopausal	40	8	0.289	1.638	0.659–4.071
WBC (x 10^12^ cells/L)	5.97±1.47	6.60±1.54	0.088	1.315	0.960–1.802
Platelet (x 10^12^ cells/L)	262.1±62.8	270.7±58.0	0.573	1.002	0.995–1.010
Hemoglobin (g/dL)	13.2±1.3	12.9±1.5	0.321	0.850	0.616–1.172
NLR	1.89±0.91	1.71±0.53	0.383	0.741	0.379–1.452
PLR	141.2±50.5	124.7±34.7	0.161	0.991	0.978–1.004
pT stage^‡^					
T1	89	1			
T2 + T3	45	18	0.001	35.600	4.604–272.251
Lymph node metastasis					
Absence	99	8	-	-	-
Presence	35	11	0.007	3.889	1.447–10.456
ER status					
Positive	33	16	-	-	-
Negative	101	3	<0.001	16.323	4.474–59.550
PR status					
Positive	34	14	-	-	-
Negative	100	5	<0.001	8.235	2.761–24.561
HER2 status					
Negative	102	11			
Positive	32	8	0.112	2.093	0.842–5.205
Ki67 index status^†^					
<14%	50	3	-	-	-
≥ 14%	77	16	0.058	3.463	0.960–12.499
Tumor involvement of resection margin					
Negative	133	17	-	-	-
Positive	1	2	0.028	15.647	1.346–181.862
Nuclear grade					
Grade 1+2	89	4	-	-	-
Grade 3	45	15	0.001	7.417	2.326–23.652
Histologic grade					
Grade 1+2	92	4	-	-	-
Grade 3	42	15	<0.001	8.214	2.571–26.249
Primary tumor SUVmax	6.0±4.0	10.9±6.1	<0.001	1.210	1.098–1.333
Spleen SUVmean	1.90±0.21	2.62±0.31	0.004	25.321	2.807–228.393
BM SUVmean	2.06±0.32	2.16±0.42	0.212	2.400	0.607–9.487
Liver SUVmean	2.49±0.26	2.62±0.31	0.062	5.781	0.916–36.481

*BMI* body mass index, *WBC* whole blood cell counts, *NLR* neutrophil-to-lymphocyte ratio, *PLR* platelet-to-lymphocyte ratio, *ER* estrogen receptor, *PR* progesterone receptor, *HER2* human epidermal growth factor receptor 2, *BM* bone marrow

^‡^ Pathologic staging according to the 7^th^ AJCC

^†^ Ki67 value for 146 patients

### DFS analysis

**Fig 3:**
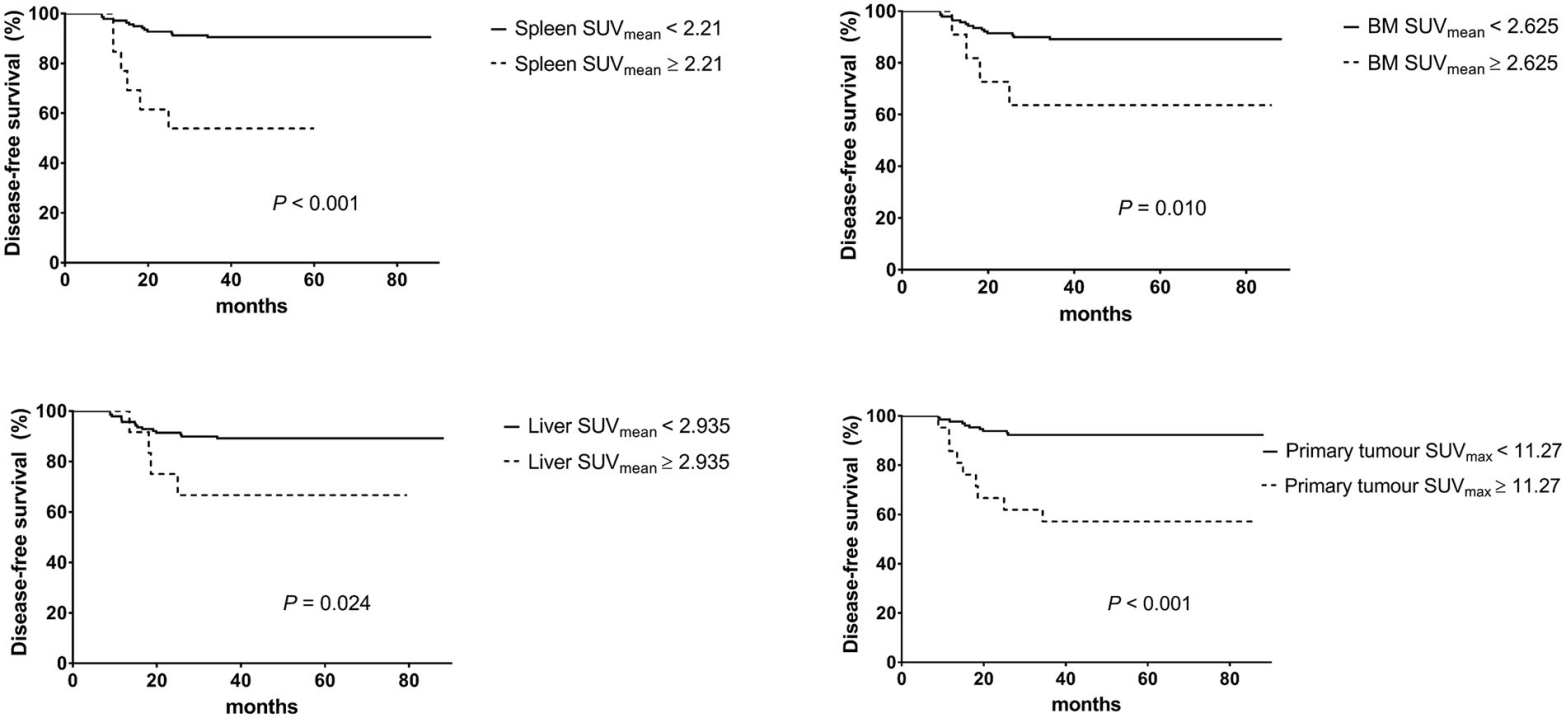
Kaplan-Meier survival curves for disease-free survival. Recurrence survival curve according to (A) spleen standardized uptake value (SUV)_mean_ cutoff at 2.21; (B) BM SUV_mean_ cutoff at 2.625; (C) liver SUV_mean_ cutoff at 2.935; and (D) primary tumor SUV_max_ cutoff at 11.27.

Univariate Cox regression analysis of DFS was performed to evaluate the prognostic potential of clinicopathological and PET parameters (Table 3). For clinical parameters, BMI and WBC were significantly associated with DFS (*P*<0.05). For pathologic parameters, pT stage, lymph node metastasis, ER/PR/HER2 status, tumor involvement of resection margin, nuclear grade, and histologic grade were significantly associated with DFS (*P*<0.05). For PET parameters, spleen SUV_mean_, BM SUV_mean_, liver SUV_mean_, and primary tumor SUV_max_ were significant prognostic factors for DFS (*P*<0.05, Fig 3).

**Table 3:** Univariate Cox regression analysis of disease free survival.

Variable	P value	HR	95% CI
Age at diagnosis	0.810	0.994	0.948–1.042
BMI (<25.0 vs. ≥25.0)	0.020	2.78	1.130–6.840
Menopausal status (pre vs. post)	0.289	1.638	0.659–4.071
WBC (< 6.39 x 10^12^ cells/L vs. ≥ 6.39 x 10^12^ cells/L)	0.022	2.84	1.120–7.210
Platelet (<348 x 10^12^ cells/L vs. ≥ 348 x 10^12^ cells/L)	0.240	2.05	0.600–7.030
Hb (<14.5 g/dL vs. ≥ 14.5 g/dL)	0.288	0.041	0.000–15.070
NLR (<2.505 vs. ≥ 2.505)	0.256	0.039	0.000–10.474
PLR (<140.7 vs. ≥ 140.7)	0.051	0.293	0.085–1.005
pT stage^†^ (T1 vs. T2+T3)	0.001	30.100	4.016–225.582
Lymph node metastasis (absence vs. presence)	0.007	3.523	1.416–8.761
ER status (positive vs. negative)	<0.001	13.754	4.003–47.256
PR status (positive vs. negative)	<0.001	7.487	2.694–20.804
HER2 status (positive vs. negative)	0.112	0.478	0.192–1.188
Ki67 index status (<14% vs. ≥ 14%)^‡^	0.051	3.408	0.993–11.700
Tumor involvement of resection margin (negative vs. positive)	0.003	9.057	2.080–39.430
Nuclear grade (Grade 1+2 vs. 3)	0.001	6.634	2.200–20.001
Histologic grade (Grade 1+2 vs. 3)	<0.001	7.290	2.418–21.982
Primary tumor SUV_max_ (< 11.27 vs. ≥ 11.27)	<0.001	6.83	2.770–16.850
Spleen SUV_mean_ (< 2.21 vs. ≥2.21)	<0.001	6.29	2.680–16.610
BM SUV_mean_ (< 2.625 vs. ≥ 2.625)	0.010	3.87	1.280–11.690
Liver SUV_mean_ (< 2.935 vs. ≥2.935)	0.024	3.33	1.100–10.030

*BMI* body mass index, *WBC* whole blood cell count, *Hb* hemoglobin, *NLR* neutrophil-to-lymphocyte ratio, *PLR* platelet-to-lymphocyte ratio, *ER* estrogen receptor, *PR* progesterone receptor, *HER2* human epidermal growth factor receptor 2, *BM* bone marrow

^‡^Ki67 value for 146 patients

^†^Pathologic staging according to the 7^th^ AJCC

Of the variables, those with a P-value less than 0.05 in univariate Cox regression analysis were selected for multivariable analysis. Among these variables, BM and liver SUV_mean_ were excluded, and only spleen SUV_mean_ was included in the multivariable analysis due to possible biological correlation among the three organs as responders to systemic inflammatory change. Multivariable analysis revealed that spleen SUV_mean_ was a factor predicting DFS independent of pT stage and ER status (*P* = 0.032, Table 4).

**Table 4:** Multivariate Cox regression analysis of disease free survival.

Variable	β	Hazard ratio	95% CI	P value
pT stage (T1 vs. T2+T3)	2.740	15.482	1.998–119.958	0.009
ER status (Positive vs. negative)	2.155	8.629	2.489–29.912	0.001
Spleen SUV_mean_ (< 2.21 vs. ≥2.21)	1.080	2.944	1.098–7.896	0.032

## Discussion

In this retrospective study, we found that spleen and BM metabolism showed a significant correlation with the tumor characteristics of breast cancer. Moreover, splenic metabolism from a pretreatment F-18 FDG PET/CT scan could potentially be used as a prognostic indicator for recurrence in breast cancer patients.

Spleen and BM SUVs were correlated with primary tumor SUV_max_, which was consistent with the findings of previous studies from breast, stomach or colorectal cancer [10, 11, 16, 21]. Primary tumor SUV_max_ is known as a prognostic factor of breast cancer [23, 27]. A recent study also revealed that inflammatory response in tumor-adjacent parenchyma could be associated with poor prognosis in breast cancer [28]. In particular, tumor necrosis factor (TNF) signaling pathway was associated with paratumoral inflammatory change in breast cancer, and this inflammatory process could be detected by imaging [28]. Cytokines such as TNF are well known pro-inflammatory cytokine associated with tumor proliferation and invasion [29]. F-18 FDG is a nonspecific tracer reflecting not only tumoral metabolism, but also an inflammatory process. As primary tumors show more aggressiveness, a more severe systemic inflammatory response could be triggered and could be detected by splenic and BM metabolism on F-18 FDG PET/CT. Although spleen, BM, and liver metabolisms were all significant predictors of recurrence in our patients, splenic metabolism could be the most powerful prognostic predictor among the three organs. In our study, due to the possible biological correlation between the metabolism of the three organs, multivariable analyses only included the spleen SUV_mean_ because splenic metabolism showed the most powerful predictive ability among the three organs in binary logistic regression and univariate analyses.

RES, formally called mononuclear phagocytic system (MPS) is a fundamental immune response mainly composed with phagocytes. The spleen, liver and BM are organs with high RES activity [30]. Previous studies revealed the measurement of metabolic activity of those organs on F-18 FDG PET/CT could be macroscopic approach to evaluate the assessment of immune activity of RES. Several studies have linked splenic metabolism with activation of a systemic inflammatory response in patients with inflammatory [13, 31] and cardiovascular diseases [32, 33]. The metabolism of the spleen has also received attention as a prognostic predictor because it is a reflector of the systemic inflammatory response to cancer [14–16]. In addition, our results indicate that splenic metabolism as a component of RES might be useful as a possible stratification parameter of F-18 FDG PET/CT in breast cancer patients.

Although the metabolisms of the spleen, BM, and liver were significant parameters in prediction of recurrence, hematologic parameters other than Hb did not correlate with metabolism of these organs. A possible explanation could be failure of NLR or PLR in predicting the recurrence of breast cancer patients in our study contrary to previous studies [17–20]. There have been controversies on the association between PLR and prognosis of breast cancer patients, possibly due to different study groups and cutoff values [18–20]. We included patients who underwent curative surgical resection without neoadjuvant therapy; therefore, the included study group might be at an earlier stage than patient groups reported previously. Furthermore, a possible explanation may be that the metabolism of the spleen, BM, or liver on F-18 FDG PET/CT could precede a change in peripheral blood cell counts.

Another consideration was the negative correlation between the metabolism of BM and Hb level. The metabolism of BM on F-18 FDG PET/CT might represent an aggregate of the complex processes of hematopoiesis, which occur not only from a systemic inflammatory response, but also in response to anemia. In this context, measuring the metabolism of the spleen could be more straightforward than the metabolism of BM in representing the systemic inflammatory response. In addition, although compromised spines due to skeletal problems were eliminated from the measurement, measuring the BM could be technically affected by the metabolic activity of bony cortex of vertebra. Although the methodology of measuring BM metabolism was established through several studies, assessing the metabolism of the spleen might be more feasible than the metabolism of BM on F-18 FDG PET/CT scans [9, 11, 24, 25]. Taken together, these findings indicate that splenic metabolism could represent a systemic inflammatory response to cancer and be used as a possible prognostic predictor in breast cancers.

A limitation of this study was the relatively small number of enrolled patients and the retrospective design from a single center. Due to a few recurrence events, there was a limitation in designing the models of multivariable analysis. A large number of patients from multiple centers with further long-term follow-up might be needed to cross-validate of our findings. In our study, enrolled patients were mainly early breast cancer, therefore, assessment of advanced breast cancer should be needed. Furthermore, study on stratification of RES metabolism among staging and molecular subtypes of breast cancer might lead to the profound understanding to RES metabolism. Moreover, other biomarkers, such as CRP, could not be assessed in this study because they were not included in routine preoperative work-up. It might be helpful to include biomarkers representing the systemic inflammatory response to differentiate the stratification of prognosis in cancer patients as a preoperative assessment.

Interestingly, the metabolisms of spleen, BM, and liver were all significantly correlated with BMI. Obesity induces a chronic low-grade systemic inflammation and is known as a risk factor of various cancers including breast cancer [34, 35]. Even though the metabolism of the liver could be affected by various factors [36–38], there has been indirect evidence of the relationship between metabolism of the liver and obesity [39]. In our study, we excluded patients who had liver disease or abnormal LFT to avoid a possible confounding effect on liver metabolism. Therefore, our results might be indirect evidence of the metabolism of the spleen, BM, and liver on F-18 FDG PET as possible representations of systemic inflammation from obesity.

Although a direct linkage between systemic inflammatory response and cancer prognosis has not been shown, and the metabolism of RES, measured from the spleen, BM, or liver on F-18 FDG PET/CT could be a mixture of complex systemic responses to cancer, our study indicates that the metabolism of spleen, BM, and liver on F-18 FDG PET/CT could be used as a prognostic predictor of breast cancer.

## Conclusion

Metabolism of the spleen, BM, and liver on F-18 FDG PET/CT might be prognostic predictors in breast cancer patients. In particular, the splenic metabolism from preoperative F-18 FDG PET/CT might be useful for predicting recurrence in breast cancer. Although further evidence is needed to connect the metabolism of the spleen, BM, or liver to the systemic inflammatory response and to cancer prognosis, the metabolism of the spleen, BM, and liver on F-18 FDG PET/CT could be used as a prognostic predictor of breast cancer. Splenic metabolism on preoperative F-18 FDG PET/CT might be a meritorious imaging factor for discriminating patients with IDC that require adjunctive therapy to prevent recurrence.

## Supporting information

S1 File Supporting data set. (PDF)
