# Supplementary material for: Clinical utility of FDG uptake within reticuloendothelial system on F-18 FDG PET/CT for prediction of tumor recurrence in breast cancer
Source: PLoS One. 2018 Dec 7;13(12):e0208861. doi: 10.1371/journal.pone.0208861 (PMC6286142; doi:10.1371/journal.pone.0208861)
Supplement: S1 File — (PDF) [file pone.0208861.s001.pdf]

Patient_nu	age_at_dia	op_name_	op_LN_nar	adj_CTx_re	RTx	HTx_code	adjCTX_do	adjTX
1	36	1	1	1	1	1	1	1
2	62	1	1	1	0	0	0	4
3	48	1	2	2	1	1	1	1
4	39	1	1	1	1	2	1	1
5	50	1	1	1	1	1	1	1
6	49	1	1	2	1	2	1	1
7	41	1	1	2	1	2	1	1
8	42	1	2	1	1	3	1	1
9	59	2	2	1	0	1	1	4
10	67	1	1	0	1	2	1	3
11	48	1	1	1	1	1	1	1
12	74	1	1	0	1	1	1	3
13	47	1	2	1	1	1	1	1
14	50	1	1	1	1	2	1	1
15	64	1	1	0	1	1	1	3
16	71	1	1	0	1	1	1	3
17	51	2	1	1	0	1	1	4
18	44	1	1	1	1	1	1	1
19	55	2	2	2	1	0	1	2
20	46	1	1	0	1	1	1	3
21	48	1	2	2	1	2	1	1
22	58	1	1	1	1	1	1	1
23	45	1	2	1	1	0	1	2
24	53	2	2	1	1	1	1	1
25	47	2	2	1	0	1	1	4
26	40	2	2	1	0	1	1	4
27	49	1	2	1	1	1	1	1
28	43	1	2	1	1	2	1	1
29	48	2	2	2	0	2	1	4
30	52	1	1	1	1	3	1	1
31	35	1	2	1	1	1	1	1
32	51	1	2	1	1	1	1	1
33	55	1	1	1	1	0	1	2
34	42	1	1	1	1	2	1	1
35	32	1	1	1	1	0	1	2
36	53	2	2	2	0	2	1	4
37	38	1	2	2	1	0	1	2
38	47	1	1	1	1	2	1	1
39	75	1	1	0	1	2	0	3
40	52	2	1	1	0	2	1	4
41	45	1	1	1	1	2	1	1
42	41	1	2	1	1	0	1	2

43	52	2	2	1	0	3	1	4
44	52	2	2	1	1	0	1	2
45	32	1	2	1	1	2	1	1
46	40	1	2	1	1	1	1	1
47	72	2	2	2	0	1	1	4
48	42	2	2	2	1	2	1	1
49	37	1	0	2	1	2	1	1
50	52	1	2	1	1	0	1	2
51	59	1	2	1	1	1	1	1
52	52	1	1	2	1	2	1	1
53	52	1	1	1	1	0	1	2
54	61	1	1	1	1	1	1	1
55	45	1	1	2	1	2	1	1
56	59	2	2	1	1	1	1	1
57	53	1	1	2	1	2	1	1
58	61	1	2	1	1	1	1	1
59	40	2	2	1	0	1	1	4
60	49	1	2	1	1	2	1	1
61	44	1	1	1	1	1	1	1
62	48	1	2	1	1	1	1	1
63	41	2	1	1	0	1	1	4
64	41	1	2	1	1	1	1	1
65	55	1	1	1	1	3	1	1
66	43	1	1	1	1	2	1	1
67	58	1	1	2	1	2	1	1
68	53	1	2	2	1	1	1	1
69	58	1	1	1	1	2	1	1
70	52	1	1	1	1	1	1	1
71	52	1	1	1	1	1	1	1
72	44	1	1	2	1	2	1	1
73	47	1	2	1	1	2	1	1
74	37	1	2	1	1	2	1	1
75	49	2	2	1	0	0	1	4
76	52	1	1	2	1	2	1	1
77	35	1	2	2	1	2	1	1
78	37	1	2	2	1	2	1	1
79	45	1	2	2	1	2	1	1
80	61	1	2	1	1	1	1	1
81	43	1	1	1	1	0	1	2
82	48	1	1	1	1	3	1	1
83	39	1	2	0	1	0	0	6
84	34	1	1	2	1	2	1	1
85	54	2	2	1	0	3	1	4

86	41	1	1	1	1	2	1	1
87	36	1	2	2	1	2	1	1
88	70	2	2	0	1	1	1	3
89	64	2	2	3	0	0	0	5
90	45	1	1	1	1	3	1	1
91	60	1	2	2	1	2	1	1
92	50	1	2	2	1	2	1	1
93	39	1	2	1	1	2	1	1
94	41	1	2	1	1	2	1	1
95	43	1	1	2	1	2	1	1
96	43	1	2	2	1	2	1	1
97	31	1	2	1	1	2	1	1
98	61	1	2	3	1	2	2	1
99	70	1	2	1	1	1	1	1
100	46	1	1	2	1	2	1	1
101	40	1	2	0	1	2	1	3
102	49	1	1	2	1	2	1	1
103	58	1	2	2	1	2	1	1
104	46	1	1	1	1	2	1	1
105	33	1	2	1	1	2	1	1
106	48	1	1	1	1	3	1	1
107	38	1	2	1	1	2	1	1
108	72	2	2	2	0	1	1	4
109	52	1	2	2	1	2	1	1
110	49	1	2	2	1	2	1	1
111	52	1	2	1	1	2	1	1
112	49	2	2	0	0	2	1	7
113	54	1	2	2	0	2	1	4
114	45	1	2	1	1	2	1	1
115	48	1	2	1	1	2	1	1
116	54	1	1	1	1	2	1	1
117	53	1	1	2	1	2	1	1
118	55	1	1	1	1	2	1	1
119	46	1	2	1	1	2	1	1
120	44	1	1	2	1	2	1	1
121	36	1	2	1	1	1	1	1
122	46	1	1	1	1	2	1	1
123	42	1	2	2	1	2	1	1
124	79	1	1	0	1	2	1	3
125	40	1	2	1	1	2	1	1
126	67	1	1	0	1	2	1	3
127	48	1	2	3	1	2	2	1
128	30	1	1	1	1	2	1	1

129	61	1	1	2	1	2	1	1
130	49	1	1	2	1	2	1	1
131	36	1	1	0	1	2	0	3
132	52	1	2	2	1	2	1	1
133	64	1	1	2	1	1	1	1
134	50	1	1	2	1	2	1	1
135	47	1	2	2	1	2	1	1
136	51	1	2	2	1	2	1	1
137	50	1	2	1	1	2	1	1
138	45	1	2	1	1	2	1	1
139	52	1	2	1	1	2	1	1
140	50	1	1	2	1	2	1	1
141	58	1	1	1	1	1	1	1
142	45	1	2	1	1	2	1	1
143	69	1	1	2	1	2	1	1
144	45	1	1	1	1	3	1	1
145	45	1	1	2	1	2	1	1
146	52	1	2	1	1	1	1	1
147	47	1	2	2	1	2	1	1
148	57	1	1	0	1	2	0	3
149	45	1	2	2	1	2	1	1
150	58	2	2	1	1	2	1	1
151	50	1	1	0	1	2	0	3
152	53	1	1	1	1	2	1	1
153	50	1	1	2	1	2	1	1

pT_numbe	pN_0orpos	pStage_nu	ER_status_	PR_status_	HER2_statu	Ki67_posit	Ki67_missi	menopaus
1	0	1	1	1	1	1	0	0
1	0	1	1	0	0	1	0	1
1	0	1	1	1	0	0	0	1
2	0	3	0	0	0	1	0	1
2	0	3	0	0	1	0	0	0
1	0	1	1	1	0	1	0	0
1	0	1	1	0	1	1	0	0
2	1	4	1	1	1	1	0	0
2	1	4	0	0	0	1	0	1
1	0	1	1	1	0	0	0	1
2	1	5	0	0	0	1	0	0
1	0	1	1	1	0	1	0	1
2	1	4	1	1	0	0	0	0
1	1	2	0	0	0	1	0	0
1	0	1	1	0	0	0	0	1
1	0	1	1	1	0	0	0	1
2	0	3	0	1	0	0	0	0
1	0	1	1	1	0	0	0	0
2	1	3	0	0	0	1	0	1
1	0	1	0	1	0	0	0	0
2	0	3	1	1	1	0	0	0
1	0	1	1	1	0	0	0	1
2	1	4	0	0	0	1	0	1
2	0	3	1	1	0	1	0	1
2	1	4	1	1	0	1	0	0
2	0	3	0	1	1	1	0	0
2	1	5	0	0	1	1	0	1
2	1	4	1	1	0	1	0	0
1	0	1	1	1	0	0	0	0
2	1	4	1	1	1	0	0	0
2	1	4	0	1	0	1	0	0
2	1	7	0	0	1	1	0	1
2	0	3	1	1	0	0	0	1
1	0	1	1	1	0	0	0	0
2	0	3	0	0	0	1	0	0
2	0	3	1	0	0	1	0	1
2	0	3	0	0	0	1	0	0
1	1	3	1	1	0	1	0	0
2	0	3	1	1	0	0	0	0
1	1	3	1	1	0	#NULL!	1	1
2	0	3	1	1	0	1	0	1
2	1	4	0	0	0	1	0	0

3	1	5	1	1	1	0	0	1
2	0	3	0	0	0	1	0	0
1	1	3	1	1	0	1	0	0
1	0	1	0	0	1	1	0	0
2	0	3	0	0	1	0	0	1
2	1	5	1	1	0	1	0	0
1	0	1	1	1	0	0	0	0
2	1	4	0	1	0	1	0	0
2	0	3	0	0	1	#NULL!	1	1
1	0	1	1	1	1	1	0	0
1	0	1	0	0	0	1	0	0
1	0	1	0	1	1	1	0	1
2	0	3	1	1	0	0	0	0
3	0	4	0	0	1	1	0	1
1	0	1	1	1	0	1	0	0
2	1	5	0	1	1	1	0	1
1	0	1	0	0	1	1	0	0
2	1	5	0	1	0	1	0	0
2	0	3	0	0	1	1	0	1
1	0	1	0	0	1	1	0	0
1	0	1	1	1	1	0	0	0
2	1	5	1	1	0	#NULL!	1	0
1	1	3	1	0	1	1	0	1
1	0	1	1	1	0	0	0	0
1	0	1	1	1	0	#NULL!	1	1
1	0	1	0	0	1	0	0	1
1	1	3	1	1	0	1	0	0
1	0	1	0	0	1	1	0	1
1	0	1	0	0	1	1	0	0
1	0	1	1	1	0	0	0	0
2	1	4	1	1	0	0	0	0
1	1	3	1	1	0	0	0	0
1	1	3	1	0	0	0	0	0
1	0	1	1	1	0	0	0	0
1	0	1	1	1	0	1	0	0
1	0	1	1	1	1	1	0	0
1	0	1	1	1	0	1	0	0
1	1	3	0	0	1	1	0	0
1	0	1	0	0	0	1	0	0
1	0	1	1	1	1	1	0	0
1	1	3	1	1	0	0	0	0
1	0	1	1	1	0	1	0	0
2	0	3	1	1	1	1	0	0

2	1	4	0	1	0	1	0	0
1	0	1	1	1	0	1	0	0
2	1	5	1	0	0	1	0	1
2	0	3	0	0	1	1	0	1
1	0	1	1	1	1	1	0	0
1	0	1	1	1	0	0	0	0
1	0	1	1	1	0	0	0	0
1	0	1	1	1	0	0	0	0
1	1	3	1	1	0	1	0	0
1	0	1	1	0	0	1	0	0
1	0	1	1	1	0	0	0	0
2	1	5	0	0	0	1	0	0
1	0	1	1	0	0	1	0	0
1	1	3	0	0	1	1	0	0
2	0	3	1	1	0	0	0	0
1	0	1	1	1	0	0	0	0
1	0	1	1	1	0	1	0	0
2	0	3	1	1	0	0	0	0
2	0	3	1	1	0	1	0	0
2	0	3	1	0	0	1	0	0
1	0	1	1	1	1	1	0	0
2	0	3	0	0	0	1	0	0
1	1	3	0	0	1	1	0	1
1	0	1	1	1	0	#NULL!	1	1
2	0	3	1	1	0	0	0	0
2	0	3	0	0	0	1	0	1
1	0	1	1	1	0	0	0	0
1	0	1	1	1	0	0	0	1
1	0	1	0	0	0	1	0	0
2	1	4	1	1	0	1	0	0
2	0	3	1	1	0	#NULL!	1	1
1	0	1	1	1	0	1	0	1
1	1	3	1	1	0	1	0	1
1	0	1	1	1	1	#NULL!	1	1
1	0	1	1	1	0	1	0	0
2	1	4	1	1	1	1	0	0
1	0	1	0	0	0	1	0	0
1	0	1	1	1	1	1	0	0
2	0	3	1	1	0	0	0	1
1	1	3	1	1	0	0	0	0
1	0	1	1	1	0	0	0	1
1	0	1	1	1	0	1	0	0
1	0	1	1	1	0	0	0	0

1	0	1	1	1	0	0	0	1
2	0	3	1	1	0	1	0	0
1	0	1	1	1	0	0	0	0
1	0	1	0	0	0	1	0	1
1	0	1	0	0	1	1	0	0
1	0	1	1	1	0	0	0	0
1	0	1	1	1	0	0	0	0
1	0	1	1	1	0	1	0	1
2	1	4	1	1	0	1	0	0
2	1	4	1	1	0	1	0	0
2	1	4	0	1	0	1	0	0
1	0	1	1	1	0	1	0	0
1	1	3	1	1	1	0	0	1
2	0	3	0	0	0	1	0	0
1	0	1	1	1	0	1	0	0
1	0	1	1	1	0	1	0	0
2	0	3	1	1	0	0	0	0
2	1	5	0	0	1	1	0	0
2	0	3	1	1	0	0	0	0
1	0	1	1	1	0	0	0	1
2	0	3	1	1	0	1	0	0
1	1	3	1	1	0	1	0	1
1	0	1	1	1	0	0	0	0
1	0	1	0	1	0	1	0	0
1	0	1	1	1	0	0	0	1

body_weig	height	BMI	recur_upda	PFS_montl	spleenmea	BM2625	primary_tumor_SUVm
55	163	20.70082	0	88.26667	0	0	4.9
58	157	23.53037	0	82.7	0	0	3.8
60	156	24.65483	0	77.7	0	0	4.8
65	158	26.0375	0	85.9	0	1	16.2
61	161	23.53304	1	19.86667	0	0	5.4
54	157	21.90758	0	82	0	0	6.1
54	164	20.07734	0	79.46667	0	1	7.4
74	164	27.51339	1	34.36667	0	0	13.7
61	159	24.12879	1	18.56667	0	0	23.28
64	149	28.82753	0	79.6	0	0	4.9
50	152	21.64127	1	9.3	0	0	10
61	153	26.05835	0	67.2	0	0	7.4
56	157	22.71898	0	29.96667	0	1	8.4
50	158	20.02884	0	80.03333	0	0	5.2
56	164	20.82094	0	79.7	0	0	1.8
57	155	23.72529	0	41.46667	0	0	5.5
62	164	23.05175	0	63.3	0	1	6.2
76	163	28.60477	0	71.23333	0	0	3.7
55	157	22.31328	1	11.56667	1	0	19.83
57	161	21.98989	0	79.13333	0	0	2
82	159	32.43543	1	24.96667	1	1	11.3
60	151	26.31464	0	72.8	0	0	3.5
54	156	22.18935	1	11.66667	0	0	6.45
71	157	28.80442	0	74.06667	0	0	11.24
77	151	33.77045	1	18.1	1	1	22.27
47	159	18.59104	0	72.1	0	0	8.8
73	147	33.78222	1	14.96667	1	1	12.75
50	159	19.7777	0	10.63333	0	0	4.3
46	154	19.39619	0	26.36667	0	0	2.1
68	165	24.97704	0	64.46667	0	1	18.3
53	170	18.3391	0	60.16667	0	1	11
58	158	23.23346	1	8.866667	0	0	4.9
49	156	20.13478	0	65	0	0	5.94
55	157	22.31328	0	67.63333	0	1	6.52
50	163	18.81892	1	15.4	0	0	7.2
55	157	22.31328	0	60	1	0	4
68	162	25.91069	1	13.5	1	0	13
63	161	24.30462	0	63.16667	0	0	2.5
48	144	23.14815	0	45.3	0	0	4.8
50	151	21.92886	0	60.33333	0	0	1.8
50	154	21.08281	0	60.3	0	0	8.5
63	159	24.9199	0	60.4	0	0	17.9

54	155	22.47659	0	80.3	0	0	5.1
62	157	25.15315	1	11.6	1	1	11.5
57	166	20.68515	0	60.3	0	0	10.1
57	163	21.45357	0	60.16667	0	0	14.4
65	151	28.50752	0	60.4	0	0	10.2
47	162	17.90886	0	60.63334	0	0	16.9
59	168	20.9042	0	56.8	0	0	3.9
65	157	26.37024	1	19.1	0	0	6.8
77	161	29.70565	0	43.16667	1	0	13.1
70	157	28.39872	0	57.13334	1	0	2.3
57	155	23.72529	0	60.5	0	0	2.3
47	136	25.4109	0	57.53333	0	0	3.7
53	158	21.23057	0	60.5	0	0	2.9
61	157	24.74745	0	38.6	0	0	10.9
66	156	27.12032	0	60.23333	0	0	4.8
50	150	22.22222	0	57.26667	0	0	7.9
49	158	19.62827	0	48.8	0	0	4.4
50	157	20.2848	0	60.23333	0	0	5.3
63	164	23.42356	0	60.33333	0	0	3.8
64	157	25.96454	0	30.33333	0	0	6.6
52	162	19.81405	0	57.7	0	0	2.91
66	161	25.46198	0	59.26667	0	0	5
49	157	19.8791	0	60.46667	0	0	2.5
70	159	27.68878	0	57.1	0	0	3.1
67	152	28.99931	0	60.6	0	0	2.3
47	153	20.07775	1	25.93333	0	0	1.5
55	158	22.03173	0	58.76667	0	0	2.8
71	155	29.55255	0	55.9	0	0	14
54	155	22.47659	0	48.43333	0	0	4.2
57	156	23.42209	0	56.13334	0	0	8.9
57	167	20.43817	0	58.1	0	0	2.6
52	163	19.57168	0	57.53333	0	0	5.1
57	160	22.26563	0	57.86667	0	0	1.7
59	156	24.24392	0	56	0	0	1.9
63	157	25.55885	0	57.56667	0	0	9.6
60	158	24.03461	0	55.33333	0	0	7
43	152	18.6115	0	57.43333	0	0	4.3
54	150	24	0	55.66667	0	0	6.8
56	168	19.84127	0	51.63334	0	0	7.5
52	154	21.92613	0	55.56667	0	0	5.1
52	167	18.64534	0	55.96667	0	0	2.1
68	168	24.09297	0	55.96667	0	0	2.7
48	149	21.62065	0	55.8	0	0	8.5

63	167	22.58955	1	14.8	0	0	2.5
46	161	17.74623	0	55.8	0	0	9.9
60	157	24.34176	0	55.26667	1	0	3
57	151	24.9989	1	8.9	0	0	14.8
63	157	25.55885	0	56.8	0	0	3.5
64	157	25.96454	0	55.43333	0	0	1.7
58	162	22.10029	0	55.8	0	0	2.3
54	165	19.83471	0	55.63334	0	0	5
53	157	21.50189	0	56.5	0	0	6.7
60	160	23.4375	0	7.833334	0	0	11.3
63	164	23.42356	0	19.36667	0	0	5.1
52	169	18.20665	0	54.56667	0	0	13.6
61	161	23.53304	0	35.36667	1	0	9.3
51	151	22.36744	0	54.13334	0	0	2.32
61	163	22.95909	0	28.76667	0	0	11.1
60	160	23.4375	0	49.96667	0	0	4.2
56	157	22.71898	0	55.4	0	0	2.8
51	146	23.92569	0	56.2	0	0	4
54	163	20.32444	0	51.76667	0	0	5.7
61	164	22.67995	0	51.26667	0	0	13.3
53	157	21.50189	0	56.13334	0	0	2.8
54	161	20.83253	0	49.83333	0	0	1.8
51	159	20.17325	0	55.33333	0	0	10.7
55	150	24.44445	0	54.96667	0	0	3.2
62	164	23.05175	0	54.33333	0	0	2.7
65	164	24.16716	1	16.53333	0	0	9.2
55	150	24.44445	0	50.76667	0	0	2.4
65	162	24.76757	0	54.66667	0	0	3.3
75	150	33.33333	0	51.3	0	0	17
74	152	32.02909	0	54.86667	0	0	2.6
53	156	21.77844	0	51.76667	0	0	2.8
55	154	23.1911	0	54.96667	0	0	7.1
58	163	21.82995	0	54.56667	0	0	3.77
63	150	28	0	54.7	0	0	13.3
70	161	27.00513	0	54.26667	0	0	4.3
52	162	19.81405	0	49.93333	0	0	4.7
58	158	23.23346	0	50.3	0	0	17.5
50	158	20.02884	0	50.13334	0	0	2.34
72	149	32.43097	0	50.4	0	0	6.4
54	155	22.47659	0	48.8	0	0	1.7
59	156	24.24392	0	51.2	0	0	1.6
51	153	21.78649	0	50.2	0	0	2
44	152	19.04432	0	45.6	0	0	4.87

55	162	20.95717	0	49.83333	0	0	4.06
70	169	24.50895	0	46.76667	0	0	3.15
55	167	19.72104	0	36.16667	0	0	6.03
65	154	27.40766	0	49.2	0	0	5.49
64	153	27.33991	0	12.56667	0	0	4.99
57	163	21.45357	0	43.9	0	0	1.48
58	156	23.833	0	42.83333	0	0	2.37
65	163	24.4646	0	48.96667	0	0	2.89
55	155	22.89282	0	48.1	0	0	3.36
65	161	25.07619	0	47.8	0	0	5.46
53	157	21.50189	0	43.76667	1	0	10.53
68	160	26.5625	0	45.26667	0	0	3.12
56	148	25.56611	0	46.63334	0	0	5.26
53	161	20.44674	0	43.86667	0	0	8.52
55	156	22.60026	0	44.86667	0	0	6.8
45	160	17.57813	0	46.23333	0	0	2.7
64	161	24.69041	0	45.93333	1	0	6.6
65	155	27.05515	1	25.66667	0	0	10.6
52	155	21.64412	0	36.6	0	0	4.6
52	152	22.50693	0	45.06667	0	0	2.2
40	160	15.625	0	43.06667	0	0	7.3
55	151	24.12175	0	44.06667	0	0	11.2
55	158	22.03173	0	43.83333	0	0	9.4
68	162	25.91069	0	44.2	0	0	5.04
56	153	23.92242	0	40.56667	0	0	8.8

ıax
